# Improving CNNs classification with pathologist-based expertise: the renal cell carcinoma case study

**DOI:** 10.1038/s41598-023-42847-y

**Published:** 2023-09-23

**Authors:** Francesco Ponzio, Xavier Descombes, Damien Ambrosetti

**Affiliations:** 1 Interuniversity Department of Regional and Urban Studies and Planning, Politecnico di Torino, Turin, Italy; 2 Université Côte d’Azur/INRIA/CNRS, Sophia Antipolis, France; 3 Department of Pathology, CHU Nice, Université Côte d’Azur, Nice, France

**Keywords:** Cancer imaging, Cancer, Medical research, Biomedical engineering

## Abstract

The prognosis of renal cell carcinoma (RCC) malignant neoplasms deeply relies on an accurate determination of the histological subtype, which currently involves the light microscopy visual analysis of histological slides, considering notably tumor architecture and cytology. RCC subtyping is therefore a time-consuming and tedious process, sometimes requiring expert review, with great impact on diagnosis, prognosis and treatment of RCC neoplasms. In this study, we investigate the automatic RCC subtyping classification of 91 patients, diagnosed with clear cell RCC, papillary RCC, chromophobe RCC, or renal oncocytoma, through deep learning based methodologies. We show how the classification performance of several state-of-the-art Convolutional Neural Networks (CNNs) are perfectible among the different RCC subtypes. Thus, we introduce a new classification model leveraging a combination of supervised deep learning models (specifically CNNs) and pathologist’s expertise, giving birth to a hybrid approach that we termed *ExpertDeepTree* (ExpertDT). Our findings prove ExpertDT’s superior capability in the RCC subtyping task, with respect to traditional CNNs, and suggest that introducing some expert-based knowledge into deep learning models may be a valuable solution for complex classification cases.

## Introduction

**Figure 1:**
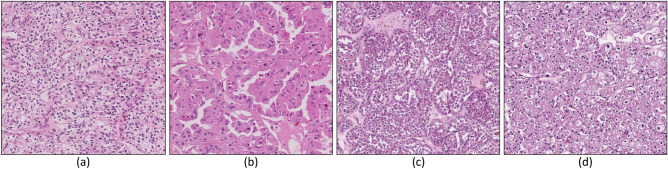
H &E samples of RCC neoplasm subtypes. The figure shows four different histological categories of RCC neoplasm subtypes: (**a**) clear cell RCC, ccRCC; (**b**) papillary RCC, papRCC; (**c**) chromophobe, chrRCC (**d**) oncocytoma, ONCO.

Renal cell carcinomas (RCCs) are currently categorized into several different histological subtypes^1^. This categorisation mainly leverages microscopic features defined by routine light microscopy analysis, immunohistochemistry profile of protein expression, and genetic alteration. Among RCC subtypes, the three most common are clear cell (ccRCC), papillary (papRCC), and chromophobe (chrRCC), including 70% to 80%, 14% to 17%, and 4% to 8% of all RCCs, respectively^1^. Approximately 10% of renal tumors belong to the benign entities neoplasms, the most frequent corresponding subtype being oncocytoma (ONCO) (3–7% of all renal neoplasms^1,2^). Figure 1 provides representative examples of the histological aspect and the structural characteristics of the above mentioned RCC subtypes.

Pathologists describe a renal tumor with different parameters, conditioning the prognosis and management, in particular the stage, the grade and the histological subtype. The latter is certainly the most difficult to establish, subtypes being defined by microscopic morphological aspects which can overlap and cause differential diagnoses, and new entities being also regularly described.

Importantly, the outcome of RCC malignant neoplasms depends on an accurate determination of the histological subtype: clear cell RCC patients have an overall 5-year survival rate of 55–60%, whereas for papillary RCC patients, it varies from 80 to 90% and for chromophobe RCC patients, it is 90%. In addition, defining subtype of these tumors has a theranostic value, each kind of tumor having its own oncogenesis mechanism, rational for the administration of a treatment. This emphasizes the need for the most accurate subclassification^3,4^.

Furthermore, existing research recognises the critical role played by the differential diagnosis between chromophobe and oncocytoma, which is known to be difficult and prone to errors due to overlapping morphological characteristics in some cases^1,2,5^.

Currently, the assessment of microscopic features of RCC neoplasm is done by light microscopy visual analysis of Haematoxylin and Eosin (H &E) stained slides, consisting most often of physical slides and, in some centers equipped with scanner, of virtual slides, the so-called Whole Slide Images (WSIs).

Hence, RCC subtyping is a time-consuming process, sometimes requiring expert review, with great impact on diagnosis, prognosis and treatment.

Computerized methods may significantly improve the efficiency and objectiveness of microscopy RCC analysis. This might be especially true for deep learning-based (DL) methodologies, largely and successfully applied to late medical and biological research^6,7^. In this regard, a large and growing body of literature has specifically investigated Convolutional Neural Network (CNNs) in relevant digital pathology classification tasks, such as lung^8^, colon^9^, breast^10^ and prostate^11^.

In this study, we firstly show how several state-of-the-art CNNs provide perfectible performance in the classification of four different RCC subtypes (ccRCC, papRCC, chrRCC and ONCO). On top of this, we propose a novel strategy for automatic RCC subclassification, leveraging a combination of supervised DL models (CNNs) and pathologist’s expertise, and thus giving birth to a hybrid approach, referred to as *ExpertDeepTree* (ExpertDT) in the rest of the manuscript. The pathologist’s knowledge is embodied in our ExpertDT’s tree-style architecture, which is made up of three classification steps in series directly designed by the pathologist (further details will follow). The ExpertDT’s superior ability in the RCC subtyping task ultimately suggests that inserting expert-based knowledge and methodology into a deep learning framework can boost the system’s performance in very cumbersome classification cases.

## Results

### Patients cohorts

Tissue samples from 91 consecutive patients, who had undergone nephrectomy in the Nice Hospital Urology Department, diagnosed with ccRCC (n = 56), papRCC (n = 22), chrRCC (n = 6) or ONCO (n = 7), were included. As defined by the 2022 WHO criteria, the diagnosis was based on pathology and cytogenetic analysis. H &E stained WSI (scanned using a Leica AT2 Digital Slide Scanner, Leica Microsystems CMS GmbH, Wetzlar, Germany) used for diagnosis were collected to define a dataset consisting in a total of 201 WSIs. The average number of slides per patient is 2.2 with a standard deviation of 1.9. The overall distribution of tumour/non-tumour tissue among the WSIs annotated by the pathologist is 72.4/27.6% in the training set and 68.9/31.1% in the test set.

### RCC subtyping with convolutional neural networks

**Figure 2:**
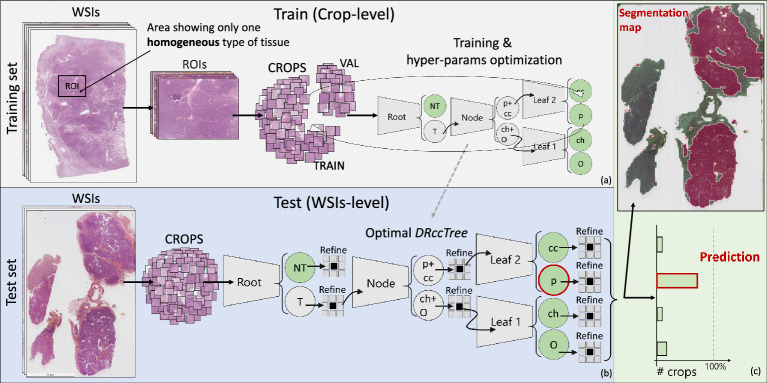
Overview of the proposed ExpertDT. The training phase is based on homogeneous regions depicting the same type of tissue, a-priori identified by a pathologist (**a**). On the contrary, the testing phase involves the whole WSI (**b**). Note that the final classification at patient-level comes from a majority voting among the predicted tiles associated to the same subject (**c**).

In Table 1 we report the average accuracy score (a.k.a. balanced accuracy)^12^ on the test set at crop-level (average accuracy score ± standard deviation over the four classes of interest) obtained in our experiments concerning the canonical CNNs. The aim of this experimental validation is to identify a proper backbone for our ExpertDT, as well as to define a comparison term, as later detailed. As it can be gathered from the first column of Table 1, irrespective of the depth and architectural complexity, none of the CNNs fully trained on the RCC dataset (column *From scratch*) was able to match the accuracy of the transfer learning frameworks (columns *TL*): the accuracy of the fully trained CNNs was almost 22% lower than the transfer learning based ones. These values suggest that the fully trained CNNs were not able to build a generalizable image representation on the given training set, most probably due to the high variability of the image characteristics, the intrinsic complexity of the RCC subtyping task ^5^, and the relatively low number of patients used for training.

Despite being remarkably better, the outcomes of the transfer learning techniques, irrespective for ImageNet or CRC pre-training, were still not totally satisfactory, showing an average accuracy score equal to 78% with a standard deviation among classes of 16% (see the second and third column of Table 1 for transfer learning from ImageNet or from CRC respectively). Please note that details concerning the different transfer learning solutions will follow in the “Methods” section.

As the comparison term, we select the most performing CNN, namely VGG16 pre-trained on the ImageNet. For the selected model, referred to as *baseline* in the rest of the manuscript, we obtain, through majority voting, as average accuracy score at patient-level around 81%.

Note that all the further analyses carried on in our study leverage the patch-level predictions aggregated by majority voting to provide the average accuracy score at patient-level as a performance metric. See “Methods” section for further details.

**Table 1:** Canonical CNNs accuracy at the patient-level on the test set (average accuracy score ± std over the classes).

	From scratch	TL-ImageNet	TL-CRC
VGG16	0.51 (± 0.14)	0.78 (± 0.16)	0.68 (± 0.13)
ResNet50	0.48 (± 0.30)	0.75 (± 0.15)	0.67 (± 0.16)
ResNet101	0.47 (± 0.13)	0.69 (± 0.11)	0.67 (± 0.15)
DenseNet121	0.50 (± 0.28)	0.73 (± 0.14)	0.57 (± 0.11)
Inception Xception	0.51 (± 0.18)	0.61 (± 0.31)	0.58 (± 0.26)
ConvNeXt	0.53 (± 0.21)	0.70 (± 0.37)	0.68 (± 0.16)

### RCC subtyping with ExpertDeepTree

To improve the classification performance in our challenging RCC subtyping task, we implemented an original classification strategy termed ExpertDT. Our methodology is made up of binary CNN classifiers (see the gray trapezoids in Fig.  2b) organized in a tree-style architecture directly originating from the pathologist’s expertise (further details will follow in the “Methods” section).

**Figure 3:**
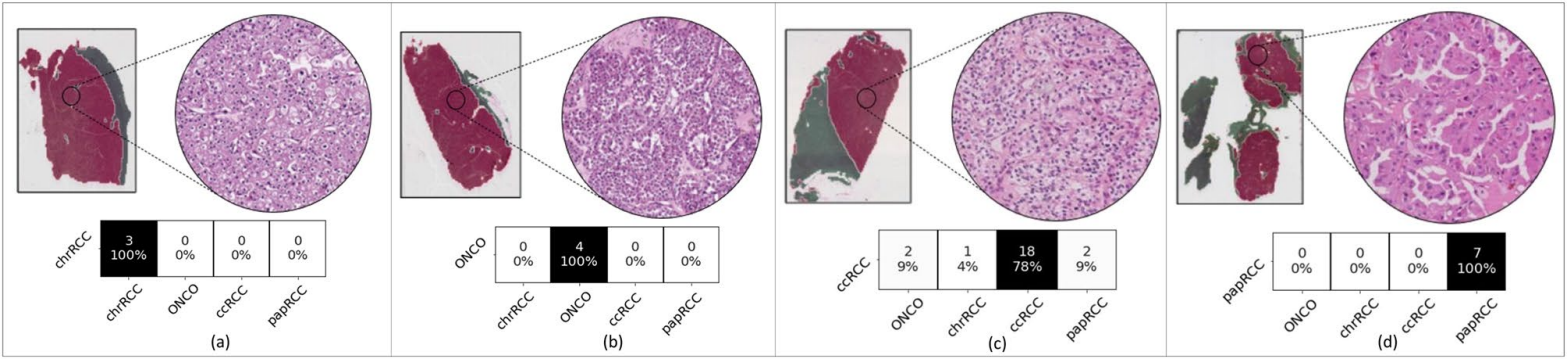
ExpertDT’s performance in the RCC subtyping task. Each box, specifically tailored to one kind of RCC subtype, (**a**) chrRCC, (**b**) ONCO, (**c**) ccRCC, (**d**) papRCC, reports: (i) an example of heatmap produced by our system (with the healthy tissue colored in green and the tumour overlapped by red); (ii) a magnification of a tissue portion belonging to the class of interest; (iii) the distribution of the classification accuracy over the different RCC subtypes.

**Figure 4:**
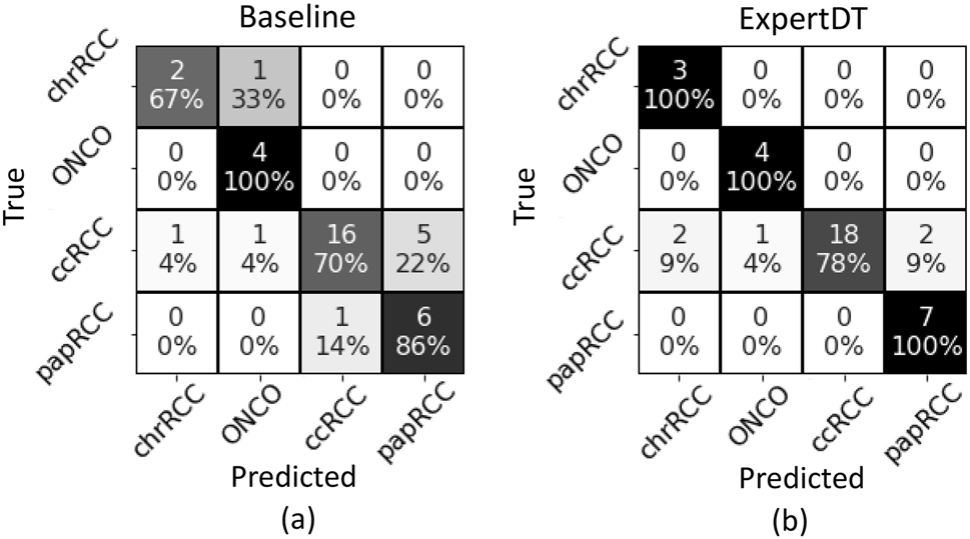
Classification performance of the baseline (left) and of the proposed ExpertDT (right) on the RCC subtyping categorization task.

As it can be seen from Fig. 3, ExpertDT correctly categorizes all the patients diagnosed with chrRCC (a), ONCO (b) and papRCC (d), proficiently managing also the cumbersome differential classification between chromophobe and oncocytoma^2,5^. Overall, ExpertDT shows an average accuracy score of $95\%$ among the four RCC subtypes, misclassifying five patients in total (all true ccRCC). The comparison between ExpertDT and the baseline (see Fig. 4) revels that our solution outperforms the canonical CNN by a value around 14% of average accuracy score (ExpertDT = 95% and baseline = 81%). Furthermore, the baseline is less effective for the differential categorization between chromophobe and oncocytoma (see chrRCC vs ONCO in Fig. 4). In absolute terms, ExpertDT misclassifies 5 patients, while the baseline 9. Lastly, as further balanced metric, we computed the Matthews correlation coefficient (MCC)^13^for both our solution (MCC = 0.8) and the baseline (MCC = 0.64).

### Naive deep trees

To assess the effectiveness of the pathologist-based ExpertDT’s architecture, we compare our solution with the two other possible versions of the tree, hereafter referred to as *NaiveDeepTree1* (NaiveDT1) and *NaiveDeepTree2* (NaiveDT2), whose structure is not related to any pathologist’s expertise. The first column of Table 2 provides the classification score (average accuracy score ± standard deviation among the classes) of ExpertDT (first line), of NaiveDT1 (second line), of NaiveDT2 (third line), and of the baseline (last line). As it can be seen, ExpertDT outperforms by a consistent margin (almost 26%) both the NaiveDT versions, the latter being consistently less accurate even than the baseline (at least by 12%).

### The effect of the *Refine* mechanism

To evaluate the effectiveness of the *Refine* stages (see “Methods” for further details), we ablated such mechanism in the models we employed. More specifically, we tested: (i) ExpertDT *with* and *without* Refine; (ii) NaiveDTs *with* and *without* Refine; (iii) the baseline *with* and *without* Refine.

From Table 2, we can do the following considerations: (i) ExpertDT largely benefits of the Refine strategy: it shows an accuracy increased by around 9%, coupled with a decreased standard deviation among classes (see first line of Table 2); (ii) the Refine provides a similar effect also on the NaiveDT models: both the versions *with* Refine results 13% more accurate than their counterparts *without* Refine; (iii) interestingly, the refine process does not have any impact on the baseline CNN, which present the same average accuracy score and standard deviation irrespective of the Refine state (see last row of Table 2).

**Table 2:** Performance comparison in the RCC subtyping task at patient-level (average accuracy score ± standard deviation over classes).

	With refine	Without refine
ExpertDT	0.95 (± 0.10)	0.86 (± 0.14)
NaiveDT1	0.69 (± 0.24)	0.56 (± 0.10)
NaiveDT2	0.67 (± 0.10)	0.54 (± 0.15)
Baseline	0.81 (± 0.13)	0.81 (± 0.13)

The first column presents the classification accuracy of ExpertDT, NaiveDTs and the baseline *with* the Refine mechanism switched on; the second column provides the accuracy of ExpertDT, NaiveDTs and the baseline *without* the Refine mechanism.

### ExpertDeepTree’s pruning

As already mentioned, the ExpertDT’s structure was decided accordingly to the pathologist’s expertise, which identified the *Node* classification as the most cumbersome task. This is mainly due to the overlapping morphological characteristics between ONCO and chRCC and between ccRCC and pRCC, as previously discussed. This aspect can be also experimentally evinced in our analysis by looking at the confusion matrix of the baseline (see Fig. 4a) where 7 over the 9 misclassified subjects are between ONCO and chRCC, and between ccRCC and pRCC. Thus, the *Node* seems to be in charge of doing the most problematic and challenging classification. To increase the reliability of the *Node*, our ExpertDT implements the following criterion: if for a given testing subject, the difference between the total amount of tiles classified as $pap + cc$ (Leaf2) and the total amount of tiles classified as $chr + ONCO$ (Leaf1) is below 30% (threshold empirically set on the training set), we do not trust the *Node*’s classification. Consequently, the *Node* is pruned and the *Root* is directly connected to the leaves.

For the sake of completeness, we implemented an ablation study comparing three different versions of ExpertDT (with and without refine): (i) the proposed ExpertDT, featuring the above described selective pruning of the *Node*; (ii) an *Unpruned* version, where the *Node* is always trust; (iii) a *Node-pruned* version, where the WSis in form of crops are fed to the *Root*, which directly triggers the downstream Leaves.

What stands out in Table 3 is the significant role played by the *Node*: when pruned, we see a large drop of classification accuracy (about 39% less accurate if compared with the original ExpertDT). Furthermore, also relying on the *Node* classification may be dangerous when the the difference between the total amount of tiles classified as $pap + cc$ and the total amount of tiles classified as $chr + ONCO$ is below 30%. This reflects the pathologist’s workflow when visually studying the morphological features of the tumors: in presence of large areas ascribable to more RCC subtypes further examinations are required (e.g. immunohistochemistry analysis).

**Table 3:** Performance comparison of the ExpertDT ablation study.

	With refine	Without refine
ExpertDT	0.95 (± 0.10)	0.86 (± 0.14)
Unpruned	0.90 (± 0.11)	0.82 (± 0.13)
Node-pruned	0.56 (± 0.19)	0.51 (± 0.20)

### Segmentation maps

Besides the classification output, our ExpertDT provides high-resolution segmentation maps highlighting the cancerous areas (see the red regions in Fig. 3). This visualisation technique allows a more in-depth analysis of the classification results, providing a tool that the pathologist can leverage to visualise the different areas of the given WSI with the corresponding predicted label.

## Discussion

In this original study, we define a strategy using deep learning based methodology to improve automated diagnosis and subclassification of RCC tumors. We demonstrate that our strategy, reproducing the stages and the decision-making algorithm of the pathologist, improves and surpasses naive global strategies.

The subtyping of RCCs is known in the literature to be a sometimes difficult task for the pathologist, often with the need to use complementary techniques. Immunohistochemistry defined phenotype and genetic anomalies provide complementary data often useful for differential diagnosis. This is for example the case of the differential diagnosis between chromophobe RCC and oncocytoma. As this task is sometimes difficult for the pathologist, it is especially challenging for artificial intelligence tools using just morphologic histopathological data to carry out classification.

In our study, we showed how several transfer learning-based and full-trained state-of-the-art CNNs provide limited classification capabilities in such categorization task. In particular, even transfer learning, traditionally a valuable strategy that can be taken into account in presence of complex classification tasks^9,14^, was not feasible in the case of RCC subtyping, where we observed reduced learning performance in the target domain.

On top of these considerations, we introduced ExpertDT, an hybrid approach between DL models and expert-based knowledge, featuring a tree-style architecture designed upon pathologist’s expertise. Our solution was able to substantially outperform canonical state-of-the-art CNNs in the classification among the four RCC subtypes. Notably, when ExpertDT is compared to comparable trees with a structure that is not based on the pathologist’s expertise, the gap of performance is even larger. These findings suggest that introducing some sort of previous expert knowledge and methodology into DL models is a valuable solution for very cumbersome classification cases. Nonetheless, relatively few studies have investigated DL applied to RCC subtyping and our work helps improving what has already been described.

Most of previous published work rely on a classification taking into account only the 2^15,16^ (or 3^3,4,17,18^) main malignant tumor subtype, ccRCC and papRCC (and chrRCC). The increase in class number not only increases the complexity of the classification process, but also pose the problem of data imbalance. This was taken into account with the development of strategies to reduce the problem of multi-class classification to several binary classification problems. This has helped to divide the multi-class classification task into several binary classification tasks which not only improved model performance, but have also helped to manage data imbalance. By taking into account more histologic subtypes, we have demonstrated the feasibility of classify approaching a daily use practice. This strategy also makes it possible to consider increasing the number of neoplasm entities under evaluation. Our ExpertDT can indeed be extended by placing the new entities in a strategic point of the decision-making tree worflow, according to the expertise of a skilled pathologist.

Previously reported works are often based on exploitation of the TCGA database^3,4,17^, without rendering results at the WSI level^3,4^ or at the patient-level^3,4,17^. In 2020, Fenstermaker et al.^3^ proposed a CNN-based strategy to classify a selection of 3486 patches retrieved from WSIs, and belonging to three classes of interest: ccRCC, papRCC and chrRCC. The authors got a patch-level accuracy up to 99%, but they do not provide the WSI-level statistics. The image database on which we carried out our study is developed from patients followed in our institution, and therefore presents a proportion of each of the entities linked to the epidemiology of these tumors. In addition, we have done our analysis by considering the WSIs with results on the patients level. These elements of our methodology are also closer to everyday practice.

Some other recent works attempt to evaluate the impact of traditional machine learning pipeline, leveraging morphological hand-crafted features to discriminate between two^15,16^ or three^18^ classes of RCC subtypes. These works are interesting to show the feasibility and improve the reliability of the classification, but these workflows and methodology do not consider the cumbersome differential diagnosis between chRCC and ONCO.

Up to our knowledge, just one previously published study by Zhu et al.^19^ investigated the categorization in more than three classes of RCC neoplasm subtypes, including the difficult oncocytoma class, as we have done. This is partially due to the relatively low frequency of oncocytoma (3–7% of all renal neoplasms^2^) and the consequent data scarcity. On the other hand, most of the previous research project exploited data gathered from TCGA data portal^20^, which is dedicated to malignant tumors, excluding renal oncocytoma cases. Even if the methodology and the results are close, there are notable differences between our work and those of the team of Zhu et al.^19^. Their study consists in designing a classification process on a surgical resection database and then to test this methodology on TCGA and biopsy database. Their workflow includes a data annotation step, with a manual ROIs definition on every WSI subsequently cropped via sliding window approach to be fed to the proposed ResNet18 classifier. In our experiment, we found normal CNNs (among which ResNet50 and ResNet101) unable to reach good classification performance at patients level on our dataset. Thus, we proposed ExpertDT, which leverages pathologist’s expertise and methodology to substantially improve the classification performance of state-of-the-art CNN models. It is interesting to note that not only our overall results are in the same range, from 77 to 100%, but also for our study and TCGA validation and biopsy validation for Zhu et al. study^19^, most of misclassification concerns ccRCC subgroup. We can assume that this is due to the fact that ccRCC, which is the most frequent tumor subtype, is also known as a tumor composed of clones and sub-clones, conferring intratumor and intertumor heterogeneity, in term of microscopic morphology but also grade, and genetic anomalies^21–23^.

As future directions, we intend to expand our dataset with other RCC cancers, including rare subtypes and classes as well as heterogeneous data. Furthermore, recent investigations suggest that *self supervised learning* methodologies may be a valuable solution to avoid the annotation step, the so-called ROI-cropping procedure^14,24–27^. As a matter of fact, this step, although useful to ensure supervised training efficiency and proper classification results, is time-consuming and incompatible with high speed use. In this regard, we intend to investigate the self supervised training of the binary CNNs classifiers, backbone of our ExpertDT.

## Methods

This retrospective study was performed with the understanding and informed consent of the subjects. All of the samples used in this study are the property of the tissue collection of the Pathology Department of the University Hospital of Nice and are declared annually to the French Health Ministry. The procedures followed were approved by the institutional review board of the University Hospital of Nice. This study was conducted in accordance with the Declaration of Helsinki.

### State-of-the-art convolutional neural networks

We want to assess the efficiency of the state-of-the-art CNNs to correctly categorize the four different subtypes of RCC neoplasms and also to define the most performing training configuration.

To answer these questions, six consolidated deep network models have been put into effect through TensorFlow framework: VGG16^28^, ResNet50^29^, ResNet101^29^, Inception Xception^30,31^, DenseNet121^32^, and ConvNeXt^33^. CNNs typically need a large amount of labeled data to learn good visual representations, while preparing large-scale labeled datasets is expensive and time-consuming, especially for medical image data^9,34^. Hence, to avoid, or at least to limit, this tedious data collection and annotation phase, some researchers take as compromise ImageNet-pretrained convolutional neural network to extract visual representations from a large set of different image types, the last training steps being performed on a reduced medical images database^9,34^.

On top of this consideration, each CNN we implemented was trained following three different learning paradigms: (i) training from scratch; (ii) transfer learning leveraging ImageNet as source domain; (iii) transfer learning leveraging a different histological dataset as source domain. In this latter experimental configuration, we exploited the pre-training on the Colorectal Cancer (CRC) classification task described in a recent study by Ponzio et al.^9^ to extract visual representations closer to our final target dataset, i.e. the RCC.

To obtain representative training and testing sets, in terms of inter-subjects and inter-class variability, we opted to randomly separate 54 patients for training and to leave 37 for testing our models, i.e. with a 60/40 ratio (see Table 4). The specimen (i.e. WSIs) selected as training set, were subsequently divided by a pathologist into regions of interest (ROIs) leveraging the so-called ROI-cropping procedure^27,35^, consisting in: (i) manually dividing each slide into ROIs that are homogeneous in terms of tissue content; (ii) manually annotating the ROIs, imposing a unique label to each tissue category; (iii) dividing the ROIs into a regular grid of tiles, that can be fed into the networks. Note that, through the above-mentioned procedure, the pathologist selected ROIs depicting several different tissue types, namely: four RCC subtypes (ccRCC, papRCC, chrRCC, ONCO) and a not-cancer *super-class* (including fiber, necrosis and normal renal parenchyma).

The tiles obtained through the ROI-cropping were subsequently divided into a training and a validation set with a 75–25% random split (see Fig. 2a), ensuring that regions coming from a single subject always belong to the same set. These sets were exploited in a threefold cross-validation fashion to find optimal hyper-parameters for the canonical CNNs as well as for our ExpertDT, as later described.

Accordingly with pathologist’s expertise^17^, the tile size has been set to $1000 \times 1000$ with a downstream scaling to $112 \times 112$. A second independent cohort of 37 RCC patients, never used during the training of the models, nor for the hyper-parameters optimization phase, were randomly selected to act as the test set for performance evaluation in terms of patient-level predictions.

As Fig. 2c suggests, for both the canonical CNNs and for our ExpertDT, the final classification at patient-level comes from a majority voting among the predicted tiles associated to the same subject.

For all the different CNN models exploited in the RCC subtyping task (VGG16^28^, ResNet50, ResNet101^29^, DenseNet121^32^, Inception Xception^30,31^, ConvNeXt^33^), and their corresponding training paradigms (training from scratch, transfer learning from ImageNet, transfer learning from CRC dataset^36^), we leveraged a grid search based on the KerasTuner package^37^ to look for the optimal configuration of the following hyper-parameters: the layer from which the fine-tuning starts (when transfer learning is employed), the learning rate and the optimizer type. Such optimization was done on a specific partition of the training set, and no patients from the test set have been considered.

In particular, we found the VGG16 model pre-trained on the ImageNet starting from the 11th layer as the optimal model. The learning rate was $1\textrm{e}{-5}$ with Adam optimizer. For all the tested models, we leveraged the original network architecture described in the corresponding paper, and we set batch size equal to 128 images. All the models were trained for at most 150 epochs, leveraging an early stopping criterion based on the training loss (loss no longer decreasing for more than 20 epochs).

### ExpertDeepTree’s training

As it can be gathered from Fig. 2b, the backbone of our ExpertDT consists of binary CNN classifiers (grey trapezoids in Fig. 2b) arranged in a *tree-style architecture*, which directly stems from the pathologist’s experience, and thus is responsible for the introduction of expert-based knowledge in our DL system.

Each binary CNN is individually trained on a reduced subset of the training dataset showing only the two labels of interest for the given binary classification task, artificially balanced via random under-sampling^38^. Specifically:

1. the *Root* CNN learns the classification between tumor (T) and not-tumor (NT). The T class includes all the cancer subtypes, while the NT class is made up of tissue identified as not-cancerous by the pathologist (fiber, necrosis and normal renal parenchyma).
2. the *Node* discriminates between the two *super-labels* $pap+cc$ and $chr+ONCO$, respectively obtained from the union of papRCC with ccRCC, and chrRCC with ONCO. The specific arrangement of the two super-classes stems from the pathologist’s expertise: it is easier to categorize between the union of ccRCC and papRCC versus the union of chrRCC and ONCO, with respect to any other super-class layout or with respect to a canonical 5 class staging. Moreover, is convenient to focus on peculiar differential diagnosis between ccRCC vs. papRCC and chrRCC vs. ONCO with respect to a task made up of more categories together. This last step is put into effect by the ExpertDT’s leaves.
3. the *Leaf1* categorizes chrRCC vs. ONCO.
4. the *Leaf2* classifies ccRCC vs. papRCC.

The optimal hyper-parameters configuration for all the CNNs backbone of ExpertDT was identified on the validation set in a twofold cross-validation fashion among non-overlapping groups of patients, following the same procedure as the one described in the previous subsection. We found again the VGG16 pre-trained on the ImageNet starting from the 11th layer as the optimal model. The learning rate was $1\textrm{e}{-5}$, Adam was the optimizer, and all the models were trained for at most 150 epochs, leveraging an early stopping criterion based on the training loss (loss no longer decreasing for more than 20 epochs). Refer to Supplementary Fig. 1 for the classification performance of the single binary classifiers backbones of the proposed ExpertDT.

**Table 4:** Patient distribution in the train and test folds among the RCC subtypes.

	ccRCC	papRCC	chrRCC	ONCO
Train	33	15	3	3
Test	23	7	3	4
Tot	56	22	6	7

### ExpertDeepTree’s testing

In our ExpertDT, each CNN model, identified in Fig. 2b by means of a grey trapezoid, is a binary classifier, whose two class predictions are represented as circles. Grey circles correspond to *branch* labels, while green circles to *leaves*. At the inference phase, a *branch* is a temporary label which leads the given testing crop $x^*$ to the subsequent classification step. The whole classification process ends when $x^*$ reaches a *leaf*, which corresponds to the final class associated with it. The final classification label, output of our ExpertDT, is provided at patient-level. Since the WSIs must be cropped into thousands of crops to be fed to the proposed architecture (see Fig. 2b on the left), the final decision at patient-level derives from a majority voting among the predicted crops associated to the same patient, and excluding those crops predicted as not-tumor (NT leaf in Fig. 2b). Note that, when a given WSI is fed to our system to be classified, the first preprocessing step is the background removal. The tiles recognised as background (see the transparent part in the WSIs reported in Fig. 2) are removed from the testing pipeline, and thus are not classified. The background removal has been carried out by simply defining an average value threshold of tile pixels to eliminate empty areas, namely where the tissue is almost absent. The corresponding threshold on the mean pixel value was empirically set to 210 on the training set.

**Figure 5:**
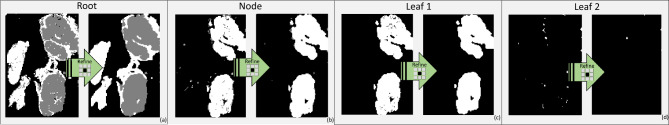
Overview of the Refine phase implemented after each classification stage of our ExpertDT (root, node and leaves). Its *low-pass, denoising* effect can be appreciated by looking at the spare black dots before and after the Refine.

### The *Refine* low-pass filtering effect

Downstream to each classification stage, we implemented the so-called *Refine* smoothing effect (see the symbol *Refine* in Fig. 2b). Such mechanism acts as a low-pass, denoising filter capable of relabelling isolated miss-classified tiles depending on the majority voting of the neighbourhood. It works as follows: for the generic testing crop $x^*$ we define: (i) its corresponding prediction $p^*$; (ii) its 9-connected neighbourhood $9N^*$, including those crops that touch either one of the edges or the corners of $x^*$ plus the pixel itself; (iii) the array of the predictions of the crops included in $9N^*$, referred to as $\vec {p^*}$.

To obtain the desired low-pass denoising effect, $p^*$ is substituted with the value obtained by the majority voting among $\vec {p^*}$. Note that, as previously mentioned, the background tiles are not fed to our model and hence not considered also in the *Refine* process. Figure 5 shows the effect of the different Refine phases implemented after each classification stage. As it can be gathered from the figure, the Refine is able to remove crops whose classification differs from its neighbourhood, which typically indicates a miss-classified crop.

### *Naive* trees

The architectures of the NaiveDT versions derive from the two other possible permutations of the *Node*’s structure: $pap+ONCO$ versus $chr+cc$ **or** $pap+chr$ versus $ONCO+cc$. Thus, they are not related to any pathologist’s expertise. For both NaiveDT1 and NaiveDT2, the *Root* CNN learns the classification between tumor (T) and not-tumor (NT). This stage is the same as in ExpertDT. Conversely, the node classification is specific for the given NaiveDT versions: $pap+ONCO$ versus $chr+cc$ for NaiveDT1, $pap+chr$ versus $ONCO+cc$ for NaiveDT2. Lastly, *Leaf1*, and *Leaf2* categorizations directly depends on the associated node: *pap* versus *ONCO* and *chr* versus *cc* for NaiveDT1; *pap* versus *chr* and *ONCO* versus *cc* for NaiveDT2.

### Supplementary Information

Supplementary Figure 1.

## Data Availability

All of the samples used in this study, property of the tissue collection of the Pathology Department of the University Hospital of Nice, are available from the corresponding author upon reasonable request and with permission of the Pathology Department of the University Hospital of Nice.
