# Supplementary material for: Improving CNNs classification with pathologist-based expertise: the renal cell carcinoma case study
Source: Sci Rep. 2023 Sep 23;13:15887. doi: 10.1038/s41598-023-42847-y (PMC10517931; doi:10.1038/s41598-023-42847-y)
Supplement: Supplementary file 1 — Supplementary Figure 1. [file 41598_2023_42847_MOESM1_ESM.pdf]

Improving CNNs classification with pathologist-based expertise: the Renal Cell Carcinoma case study

Francesco Ponzio[1,*], **Xavier Descombes**[2], **and Damien Ambrosetti**[3]

[1]Interuniversity Department of Regional and Urban Studies and Planning, Politecnico di Torino, Torino, Italy
[2]Université Côte d'Azur / INRIA / CNRS, France
[3]Department of Pathology, CHU Nice, Université Côte d'Azur, Nice, France
[*]corresponding author francesco.ponzio@polito.it

ABSTRACT

The prognosis of renal cell carcinoma (RCC) malignant neoplasms deeply relies on an accurate determination of the histological subtype, which currently involves the light microscopy visual analysis of histological slides, considering notably tumor architecture and cytology. RCC subtyping is therefore a time-consuming and tedious process, sometimes requiring expert review, with great impact on diagnosis, prognosis and treatment of RCC neoplasms. In this study, we investigate the automatic RCC subtyping classification of 91 patients, diagnosed with clear cell RCC, papillary RCC, chromophobe RCC, or renal oncocytoma, through deep learning based methodologies. We show how the classification performance of several state-of-the-art Convolutional Neural Networks (CNNs) are perfectible among the different RCC subtypes. Thus, we introduce a new classification model leveraging a combination of supervised deep learning models (specifically CNNs) and pathologist's expertise, giving birth to a hybrid approach that we termed *ExpertDeepTree* (ExpertDT). Our findings prove ExpertDT's superior capability in the RCC subtyping task, with respect to traditional CNNs, and suggest that introducing some expert-based knowledge into deep learning models may be a valuable solution for complex classification cases.

Supplementary material

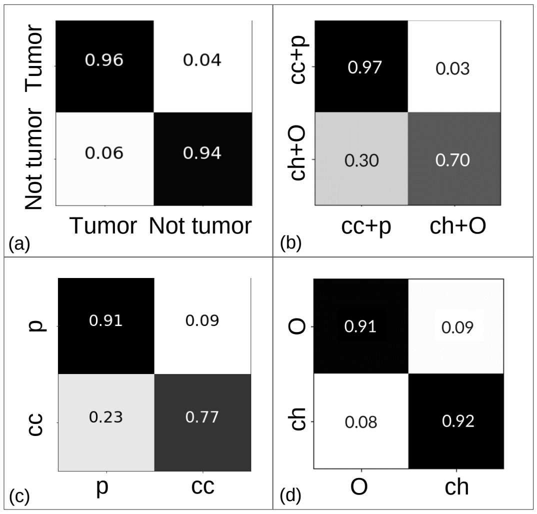

Supplementary Figure 1. Classification performance of the four binary classifiers backbones of the proposed ExpertDT: the Root (a), the Node (b), and the two Leaves (c) and (d).
